# A New Organotypic 3D Slice Culture of *Mouse* Meibomian Glands Reveals Impact of Melanocortins

**DOI:** 10.3390/ijms232314947

**Published:** 2022-11-29

**Authors:** Ingrid Zahn, Fabian Garreis, Martin Schicht, Vera Rötzer, Jens Waschke, Yuqiuhe Liu, Valerian L. Altersberger, Friedrich Paulsen, Jana Dietrich

**Affiliations:** 1 Institute of Functional and Clinical Anatomy, Friedrich-Alexander-Universität Erlangen-Nürnberg, 91054 Erlangen, Germany; 2 Department of Anatomy, Ludwig-Maximilians-Universität, 80336 Munich, Germany

**Keywords:** 3D cell culture model, dry eye disease, meibomian gland, meibomian gland dysfunction, melanocortin receptor, organotypic slice culture, vibratome, α-MSH

## Abstract

The meibomian glands (MGs) within the eyelids produce a lipid-rich secretion that forms the superficial layer of the tear film. Meibomian gland dysfunction (MGD) results in excessive evaporation of the tear film, which is the leading cause of dry eye disease (DED). To develop a research model similar to the physiological situation of MGs, we established a new 3D organotypic slice culture (OSC) of *mouse* MGs (mMGs) and investigated the effects of melanocortins on exocrine secretion. Tissue viability, lipid production and morphological changes were analyzed during a 21-day cultivation period. Subsequently, the effects on lipid production and gene expression were examined after stimulation with a melanocortin receptor (MCR) agonist, α-melanocyte-stimulating hormone (α-MSH), and/or an MCR antagonist, JNJ-10229570. The cultivation of mMGs OSCs was possible without impairment for at least seven days. Stimulation with the MCR agonists induced lipid production in a dose-dependent manner, whereas this effect was tapered with the simultaneous incubation of the MCR antagonist. The new 3D OSC model is a promising approach to study the (patho-) physiological properties of MG/MGD while reducing animal studies. Therefore, it may accelerate the search for new treatments for MGD/DED and lead to new insights, such as that melanocortins likely stimulate meibum production.

## 1. Introduction

The meibomian glands (MGs) are specialized sebaceous glands with a branched alveolar structure that traverse the eyelids [1,2]. In *humans*, about 30–40 individual MGs in the upper and 25 MGs in the lower eyelids are embedded in the tarsal plate, respectively [3]. Each individual MG consists of one central duct surrounded by 10–15 circularly arranged acini with a short connecting ductule [1]. The acini comprise secretory epithelial cells (meibocytes), which produce a lipid-rich secretion (meibum). The release follows a holocrine secretion mechanism, so that the entire cell and cell content forms the meibum [4]. To maintain this cycle of constant cell turnover, there are undifferentiated meibocytes in the periphery of the acini and differentiated meibocytes in the center towards the ductal lumen [1,5]. During this process of maturation, four stages can be distinguished by an increase in cell size, lipid droplet size and disintegration of the cell nucleus [1].

Melanocortins are protein hormones derived by the post-translational cleavage of proopiomelanocortin (POMC) in the pituitary gland [6,7]. Melanocortins include the α-melanocyte stimulating hormone (α-MSH), β-MSH and adrenocorticotropic hormone (ACTH) [8]. Most of the five identified melanocortin receptors (MC1R-MC5R) show tissue-specific expression patterns and different binding affinities for each of the melanocortins [9]. MC1R is expressed in all differentiation stages of *human* sebaceous gland cells (sebocytes) [10]. In contrast, MC5R is only detected in differentiated but not in undifferentiated sebocytes, suggesting MC5R as a sebocyte differentiation marker [11]. The sebaceous lipid production is reduced in MC5R-deficient *mice* [12], but can be upregulated by the addition of exogenous α-MSH [13,14]. In sebocytes, α-MSH upregulated lipid production as well as MC1R and MC5R expression levels [14,15]. These findings together with the fact that the pathology of acne vulgaris is associated with excessive sebum production led to the search for agents that inhibit both MC1R and MC5R as a target for acne therapy. 5-phenylamino-2, 3-bis-(2-methoxyphenyl)-1, 2, 4-thiadiazoline (free base) (JNJ-10229570) acts as an MC1R and MC5R antagonist and inhibited sebaceous lipid production in vitro and in vivo [16]. As MGs belong to the free sebaceous glands, both share similarities regarding function, secretion mode and physiology [17,18]. However, there is no information on the expression of MCRs in the MG nor on the effects of α-MSH on their differentiation and lipid production.

The meibum produced by the MGs covers the mucoaqueous component of the tear film. This outermost lipid component stabilizes and prevents excessive evaporation. Therefore, any imbalance in the functional homeostasis of the MGs will destabilize the tear film resulting in increased evaporation [19]. Hyperevaporation of the tear film leads to hyperosmolarity, which in turn induces apoptosis in conjunctival and corneal epithelial cells as well as activating the immune system triggering ocular surface inflammation [20]. These are in fact considered as the core mechanisms of dry eye disease (DED) pathology. Functional disorders of the MGs, summarized as meibomian gland dysfunction (MGD), are therefore the most common cause of evaporative dry eye (EDE), the most common subtype of DED [21,22]. There are several suggestions for the underlying pathology of MGD, such as hyperkeratinization of the central excretory ducts, androgen deficiency and aberrant peroxisome proliferative activated receptor gamma (PPARγ) traps (for a detailed review, see [23]). However, the underlying molecular mechanisms remain unclear. Therefore, intense studies on the physiological function and maturation of MG and the etiology of MGD should be conducted. To date, in vitro research on MGs and MGD is performed to a large extent using an immortalized human meibomian gland epithelial cell line (ihMGECs). This cell culture model has and will undoubtedly lead to essential findings, ideas and hypotheses, but the model has its limitations (for a detailed review see [24]). Some concerns have arisen in experiments over the past decade regarding the representation of its native function, so results should be viewed cautiously. One of the main problems is certainly that an immortalized cell line is not able to reflect the in vivo situation of a cell dying in its physiological function by holocrine secretion. Another concern is the origin of the cells from a male donor, whereas DED/EDE is known to have a higher prevalence in women than in man [4,25,26]. In addition, the lipid profile of mature ihMGECs differs from that of *human* meibum [24,27]. Nevertheless, ihMGECs provide an important contribution to research on MG/MGD [24]. However, to overcome these limitations and advance in vitro MG/MGD research to a more physiological level, the use of a newly established ex vivo MG organotypic slice culture (OSC) obtained by vibratome sectioning seems to be highly promising. With this technique, it is possible to cultivate the MG within its native tissue surrounding [28]. In this study, we aim to establish long-term cultivation for up to 21 days of this ex vivo 3D model of *mouse* MG (mMG) to investigate molecular mechanisms of MG in health and disease. We have demonstrated that cultivation is possible even up to seven/twenty-one days and functional experiments, e.g., on the influence of MCRs, can be performed. With a robust and reliable 3D model, we will be able to elucidate treatment strategies for MGD and thus for patients who suffer from DED.

## 2. Results

### 2.1. Long-Term Maintanance of Mouse Meibomian Gland Organotypic Slice Culture Viability

**Figure 1:**
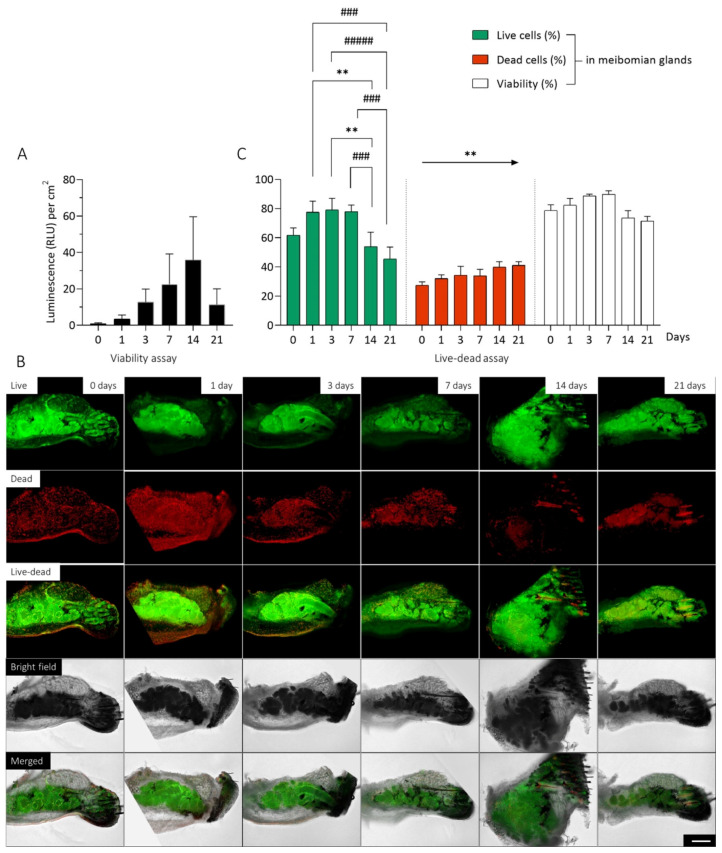
Viability of organotypic slice cultures (OSC) of *mouse* meibomian glands (mMG) over 21 days. The viability of mMG OSCs were investigated over 21 days. No decrease in viability was observed within the first 14 days of cultivation (**A**). Representative pictures of OSCs after live-dead staining illustrates the location and distribution of the live (green) and dead (red) cells. Bright field imaging shows the position of the mMGs within the OSC (**B**). The evaluation of live-dead images based on the proportion of live and dead cells and the viability of mMG during the cultivation period (**C**). Most of the live cells are located in the meibomian glands, while the dead cells are homogeneously distributed throughout the tissue. Data are n = 9 (**A**) and 4 (**B**,**C**), mean ± SEM. Significant changes compared to day 14 are assigned as */compared to day 21 are assigned as #. An arrow indicates a linear trend test. ** *p* ≤ 0.01, ### *p* ≤ 0.001, ##### *p* ≤ 0.0001 (**C**). Scale bar: 200 µm (**B**).

In order to perform reliable long-term studies, it is necessary that the OSC maintain their viability. Therefore, we examined a CellTiter-Glo 3D viability assay and a live/dead assay directly after sectioning and up to 21 days. The thickness of each OSC is constant at 150 µm, but the surface area varies. Thus, the measured luminescence (RLU) of the viability assay is normalized to the surface of each OSC. The viability increased continuously from day 0 (0.9270 ± 0.4117 RLU per cm^2^, n = 8) up to day 14 (35.83 ± 23.81 RLU per cm^2^, n = 8). After 21 days (11.32 ± 8.698 RLU per cm^2^, n = 8), the average viability decreased (Figure 1A). Treatment of OSCs with 40% DMSO resulted in a considerable decrease in viability on all days (Figure S1A).

The live/dead assay additionally illustrates the location and distribution of dead (red) and live (green) cells by immunofluorescence signal (Figure 1B). The position of the MG within the OSC can be determined using bright field imaging (Figure 1B Bright Field). There, the cilia, meibomian glands, conjunctiva, tarsus and orbicularis oculi muscle can be identified (Figure S2). The superimposition of bright field images with live/dead staining images shows the distribution of live and dead cells in the MG compared to the surrounding tissue (Figure 1B Merged) Evaluation of the area distribution of the MG compared to the surrounding tissue revealed that on average 19.07 ± 5.005% (n = 24) of the surface area of the OSC consists of MGs. In contrast, the MGs account for an average of 34.83 ± 8.266% (n = 24) of the dead cells and as much as 65.99 ± 18.71% (n = 24) of the live cells. Thus, the proportion of the area of MGs within the tissue is significantly smaller than the proportion of live (mean difference: 46.92%, *p* < 0.0001) and dead cells (mean difference: 15.75%, *p* < 0.0001), while MGs account for a significantly larger proportion in the tissue of live cells compared to dead cells (mean difference: 31.16%, *p* < 0.0001). This indicates that most of the live cells are located in the MGs, while the dead cells are homogeneously distributed throughout the tissue. During the cultivation period, there was a significant decrease in the percentage of live mMG cells in the OCS after 14 (mean difference: 24.01%, *p* = 0.0080) and 21 days (mean difference: 32.43%, *p* = 0.0001) compared to 7 days (Figure 1C Live Cells). The proportion of dead cells increased significantly (slope: 2.619, *p* = 0.0036) linearly throughout the cultivation period (Figure 1C Dead Cells). No significant difference in viability was observed within the mMGs, but there was a trend toward a decrease in viability after 14 (73.54 ± 10.15%, n = 4) and 21 days (71.54 ± 6.574%, n = 4) compared to 7 days (89.76 ± 4.925%, n = 4) (Figure 1C Viability). Treatment of OSCs with 40% DMSO resulted in little to no live cells, but there were dead cells throughout the tissue at each cultivation point. (Figure S1B).

### 2.2. Long-Term Maintenance of Lipid Secretion in Mouse Meibomian Gland Organotypic Slice Culture

**Figure 2:**
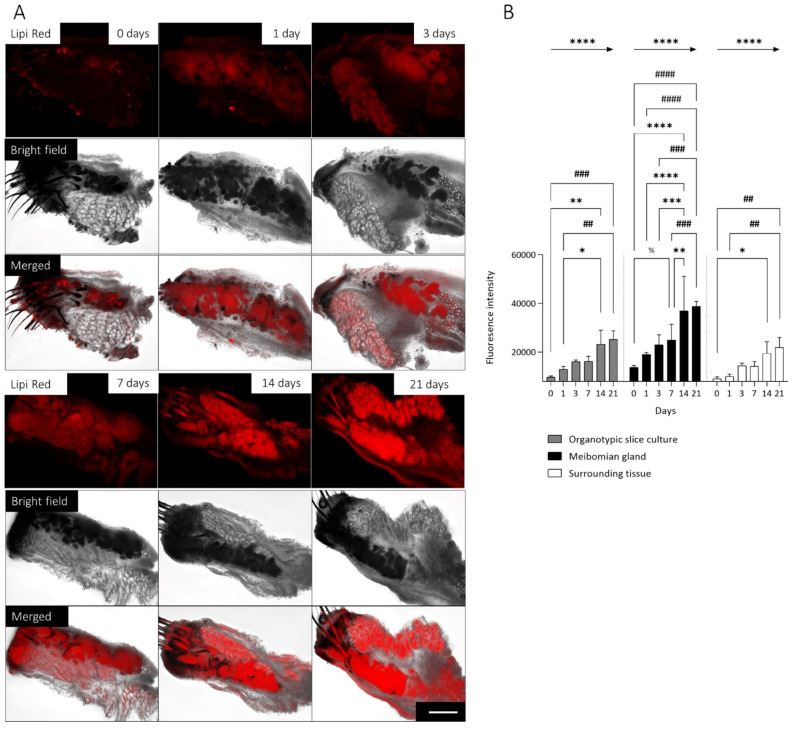
Lipid production in organotypic slice cultures (OSC) of *mouse* meibomian glands (mMG) over 21 days. Representative pictures of lipid droplets visualized by Lipi Red staining of mMG OSCs for 21 days. Bright field imaging shows the position of the mMGs within the OSC. Most lipid droplets are located in the mMGs (**A**). During the cultivation period, the fluorescence intensity increases significant linearly in the whole OSC, for the mMG as well as the surrounding tissue (**B**). Significant changes compared to day 7 are assigned as %/day 14 are assigned as */day 21 are assigned as #. Arrows indicate a linear trend test. Data are n = 4, mean ± SD, */% *p* ≤ 0.05, **/## *p* ≤ 0.01, ***/### *p* ≤ 0.001, ****/#### *p* ≤ 0.0001 (**B**). Scale bar: 200 µm (**A**).

In addition to viability, it is also important that the physiological function of lipid production is maintained in the mMGs. For this reason, we stained the OSCs with Lipi Red, which allows monitoring lipid droplets in live cells without cytotoxic effects [29]. The position of the MG within the OSC was determined using bright field imaging (Figure 2A Bright Field), and the lipid production was tracked over time (Figure 2A Lipi Red). Superimposition of bright field images with Lipi Red staining images shows the distribution of the lipids within the MG compared to the surrounding tissue (Figure 2A Merged). Within the OSC fluorescence intensity was significantly higher (mean difference: 11,204, n = 24, *p* < 0.0001) in the MGs than in the surrounding tissue. Evaluation of lipid production in the whole OSC showed a significant increase after 14 (mean difference: 13,448, n = 4, *p* = 0.002) and 21 days (mean difference: 15,339, n = 4, *p* = 0.0047) compared to directly after sectioning (day 0) and a significant increase after 14 (mean difference: 10,346, n = 4, *p* = 0.002) and 21 days (mean difference: 12,237, n = 4, *p* = 0.0047) compared to 1 day (Figure 2B Organotypic slice culture). However, in MGs, fluorescence intensity increased already significantly after 7 days compared to directly after sectioning (mean difference: 11,183, n = 4, *p* = 0.012). After 14 days lipid intensity was significantly increased compared to 0 days (mean difference: 23,133, n = 4, *p* < 0.0001), 1 day (mean difference: 17,916, n = 4, *p* < 0.0001), 3 days (mean difference: 13,948, n = 4, *p* < 0.0009) and 7 days (mean difference: 11,950, n = 4, *p* = 0.0061). In addition, lipid intensity within the MGs increased significantly after 21 days compared to 0 days (mean difference: 24,988, n = 4, *p* < 0.0001), 1 day (mean difference: 19,771, n = 4, *p* < 0.0001), 3 days (mean difference: 15,803, n = 4, *p* < 0.0001) and 7 days (mean difference: 13,805, n = 4, *p* = 0.0010) (Figure 2B Meibomian Gland). In comparison, lipid intensity in the surrounding tissue increased only after 14 days (mean difference: 10,367, n = 4, *p* = 0.0249) and 21 days (mean difference: 12,850, n = 4, *p* = 0.0026) compared to 0 days, and after 21 days (mean difference: 11,976, n = 4, *p* = 0.0060) compared to 1 days (Figure 2B Surrounding Tissue). During the entire cultivation period, fluorescence intensity increased significantly linearly both in the MGs (slope: 5162, *p* < 0.0001), the whole OSC (slope: 3082, *p* < 0.0001), and the surrounding tissue (slope: 2642, *p* < 0.0001). Treatment of OSCs with 40% DMSO as a negative control resulted in decreased fluorescence intensity after day 14 and day 21 in the whole OSC and MG (Figure S3).

### 2.3. Long-Term Preservation of Mouse Meibomian Gland Organotypic Slice Culture Morphology

**Figure 3:**
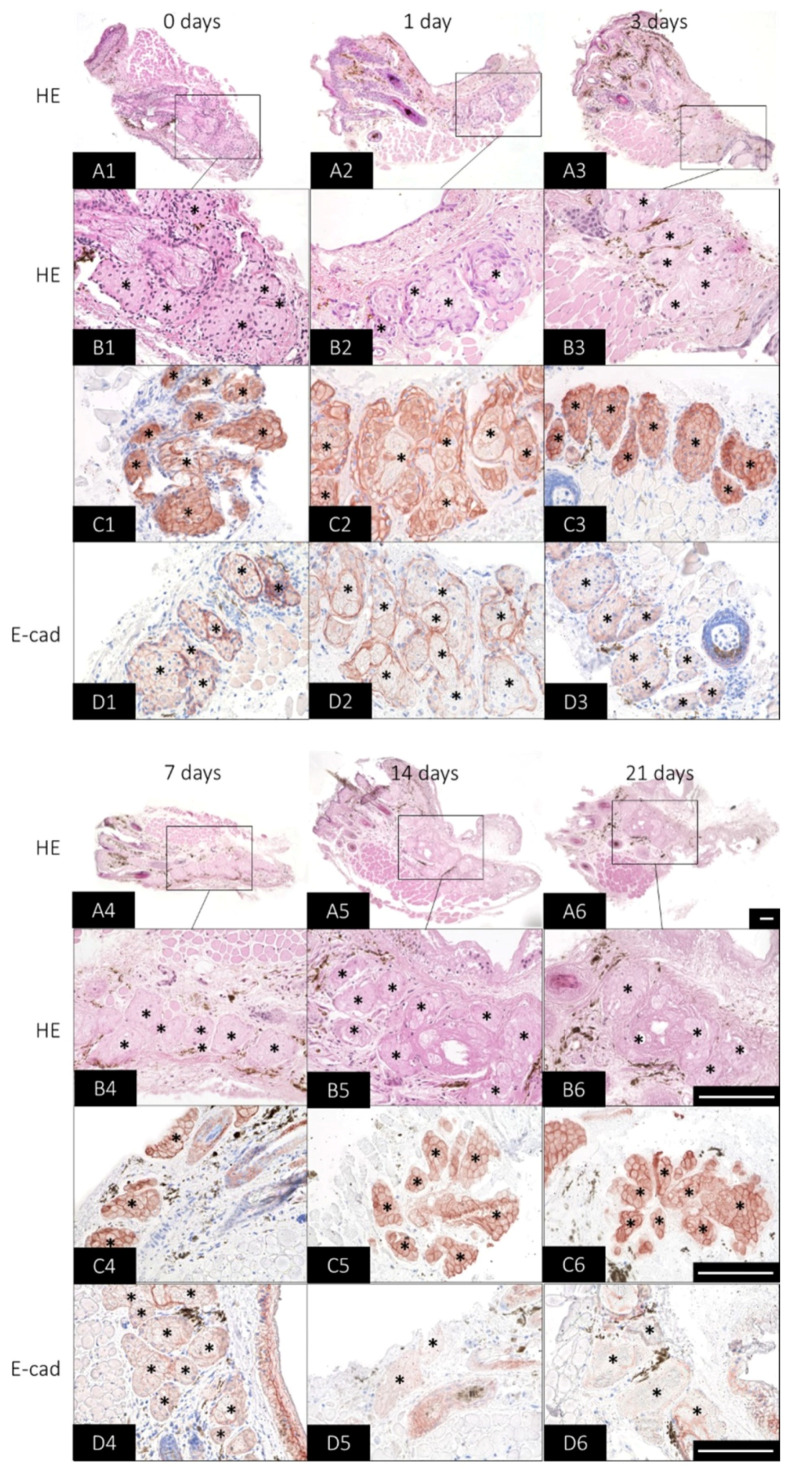
Histological changes of organotypic slice cultures (OSC) of *mouse* meibomian glands (mMG) over 21 days. Representative pictures of mMG OSC morphology visualized by hematoxylin and eosin (HE), cytokeratin 14 (CK14) and E-cadherin (E-cad)-staining for 21 days. HE at 100× magnification serves as an overview staining for the OSC (**A1**–**A6**). The 400× magnification shows that as the cultivation time progresses, fewer nuclei can be stained in the mMG acini (*) (**B1**–**B6**). CK14 antibody reaction is maintained in the meibomian gland acini (*) over the culture period (**C1**–**C6**), whereas E-cad immunoreaction decreases at 14 and 21 days (**D1**–**D6**). Data are n = 3, scale bar = 100 µm (**A1**–**D6**).

In addition to viability and physiological function, it is also of interest that the morphology is maintained over time. An overview staining of the mMG OSC revealed that there are no apparent structural changes up to 21 days (Figure 3(A1–A6)). However, after 14 and 21 days, slight tissue degeneration was observed, which was already evident during handling (Figure 3(A5–A6)). Thus, the OSCs showed occasional detachment from the surrounding low-melting-agarose. A detailed examination of the mMG acini showed that with increasing cultivation time, fewer cell nuclei could be stained by hematoxylin (Figure 3(B1–B6)). The number of stained nuclei decreased distinctly already after 3 days (Figure 3(B3)) and after 14 and 21 days hardly any nuclei were visible (Figure 3(B5,B6)).

Cytokeratin (CK) 14 forms together with CK5 intermediate filaments of epithelial cells and is considered to be an MG epithelial cell marker [30,31]. In the OSCs, CK14 antibody reaction was observed in basal, differentiating and mature meibocytes directly after sectioning and as late as 21 days after (Figure 3(C1–C6)). E-cad belongs to adherens junctions, which connect epithelial cells and are critical for epithelial development, remodeling and physiology [32]. In the OSCs, E-cad immunoreactivity was observed between basal, differentiating and mature meibocytes directly after sectioning and up to 7 days, but has a higher expression in basal cells. (Figure 3(D1–D4)). However, after 14 and 21 days, E-cad reactivity was only redundantly present (Figure 3(D5,D6)).

**Figure 4:**
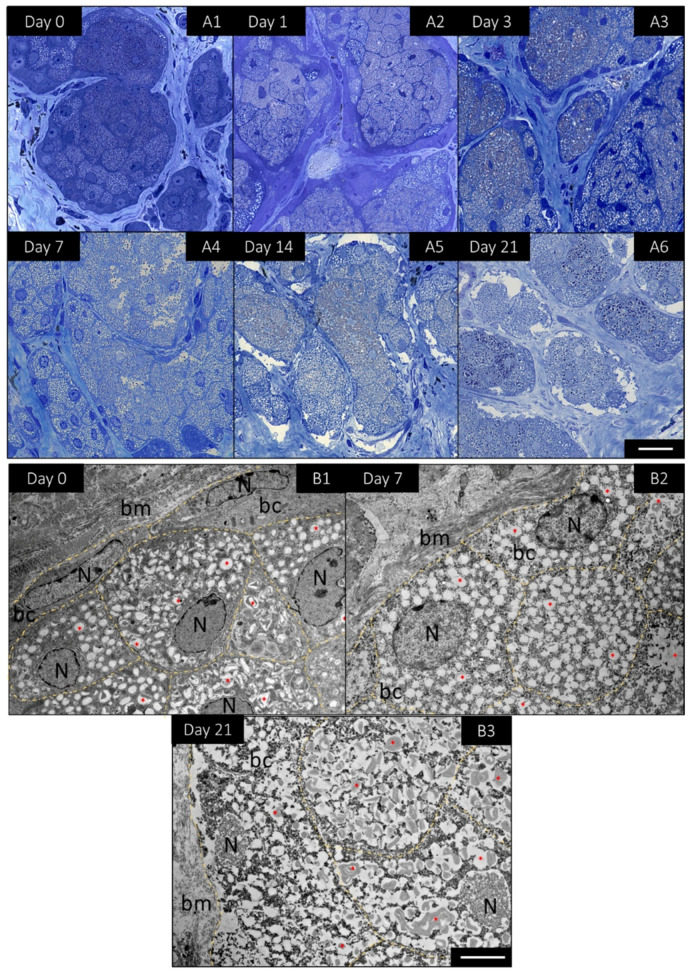
Detailed morphological changes of *mouse* meibomian gland (mMG) acini in organotypic slice cultures (OSC) over 21 days. Representative pictures of toluidine blue stained semi-thin sections of mMG OSCs for 21 days (**A1**–**A6**). Ultrathin sections analyzed by transmission electron microscopy revealed that basal cells (bc) located next to the basal membrane (bm) enlarge and undergo lipogenesis with increasing cultivation time. Thereby, the bc accumulate more lipids (*) and the nucleus (N) becomes increasingly pyknotic (day 0, day 7 and day 21) (**B1**–**B3**). Data are n = 3, Scale bar = 25 µm (**A1**–**A6**), 5 µm (**B1**–**B3**).

Semi-thin sections were prepared to visualize the changes in the acini and in particular in the basal meibocytes. HE staining had already demonstrated that fewer nuclei could be stained with increasing cultivation time. The evaluation of Toluidine blue staining of semi thin sections now revealed that the nuclei were still present in the basal meibocytes but became pyknotic over time (Figure 4(A1–A6)). In order to visualize the changes at the cellular level, representative images of basal cells were taken at 0 days, 7 days and 21 days using transmission electron microscopy. The basal cells located next to the basal membrane did not contain lipids at day 0 (Figure 4(B1)), but were enlarged and displayed rich cytoplasmic lipid droplets and the first signs of a pyknotic nucleus at day 7 (Figure 4(B2)). The same was observed after 21 days, where the basal cells were even more enlarged, lipogenesis had progressed and the nucleus was pyknotic (Figure 4(B3)). It seemed that the basal cells underwent an ongoing differentiation during day 0 and day 21 by the increase of lipid droplets and a pyknotic nuclei. Strikingly, the amount of normal looking basal cells decreased over time. The decrease in basal cells over time was accompanied by a decrease in intact nuclei, meaning that significantly fewer nuclei could be stained as cultivation progressed. Overall, the investigations of long-term cultivation show that the mMG can be maintained for at least 7 days, with only minor functional and morphological changes. Therefore, the following functional studies were performed for up to 7 days.

### 2.4. Influence of Melanocortins and Their Receptors on Lipid Production

**Figure 5:**
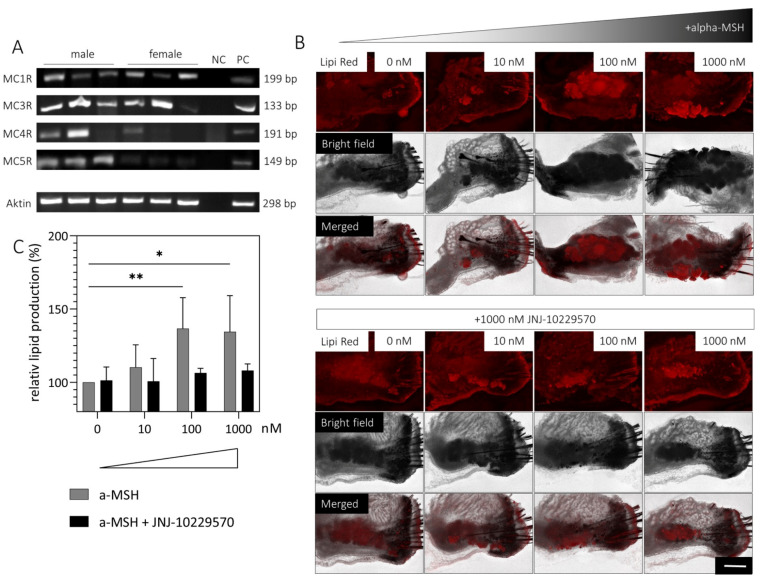
Influence of a melanocortin receptor (MCR) agonist (α-MSH) and MCR antagonist (JNJ-10229570) on lipid production in *mouse* meibomian gland (mMG) organotypic slice culture (OSC). RT-PCR detection of *MC1R*, *MC3R*, *MC4R* and *MC5R* mRNA in male and female mMG from three different *mice* (**A**). Representative pictures of lipid droplets visualized by Lipi Red staining of OSCs treated with medium containing up to 1000 nM α-MSH with/without 1000 nM JNJ-10229570 for 1 day (**B**). Lipid production increases significantly with the addition of 100 nM and 1000 nM α-MSH, whereas this effect is absent when JNJ-10229570 is added (**C**). Data are n = 4, mean ± SD, NC = negative control, PC = positive control (*mouse* brain). Scale bar: 200 µm * *p* ≤ 0.05, ** *p* ≤ 0.01.

Since there is no information on the expression of MCRs in the MGs, gene expression was investigated in male and female mMGs. An analysis showed that *MC1R*, *MC3R*, *MC4R* and *MC5R* are expressed in both male and female mMGs (Figure 5A).

The OSCs of four different *mice* (2 male/2 female) were treated with medium containing up to 1000 nM MCR agonist (α-MSH) with/without 1000 nM MCR antagonist (JNJ-10229570). After 1, 3 and 7 days, the OSCs were stained with Lipi Red and the lipid intensity was evaluated. After one day, a significant increase in lipid intensity was observed when the OSCs were stimulated with 100 nM (mean difference: 36.68, *p* = 0.0071) or 1000 nM α-MSH (mean difference: 34.44, *p* = 0.0119). However, this effect was absent when the OSCs were simultaneously incubated with 1000 nM JNJ-10229570 (Figure 5B/C). Moreover, lipid intensity tended to be increased already with 10 nM α-MSH treatment (Figure 5C). In contrast, no increase in lipid intensity was observed by the addition of α-MSH after 3 and 7 days (Figure S4).

### 2.5. Influence of Melanocortins and Their Receptors on Gene Expresssion Levels

**Figure 6:**
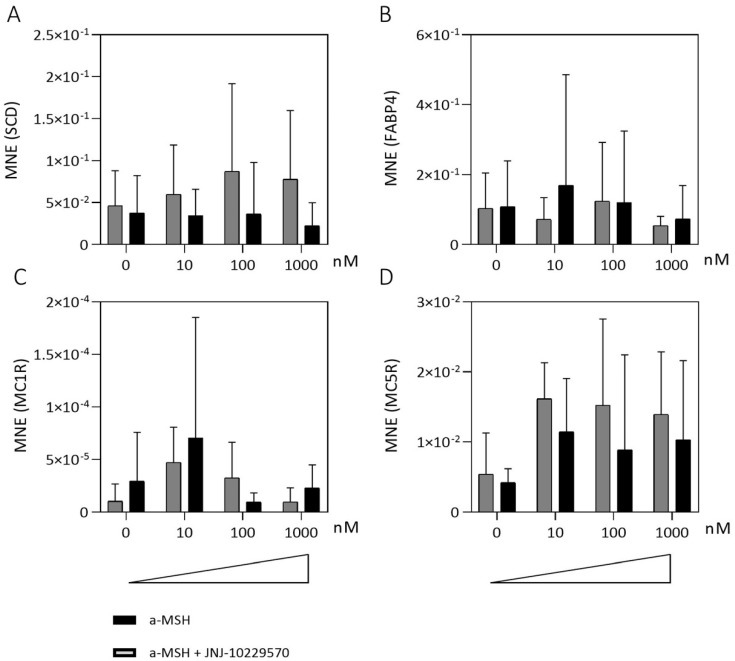
Influence of a melanocortin receptor (MCR) agonist (α-MSH) and MCR antagonist (JNJ-10229570) on gene expression levels in *mouse* meibomian gland (mMG) organotypic slice culture (OSC). Gene expression levels of OSCs treated with medium containing up to 1000 nM α-MSH with/without 1000 nM JNJ-10229570 for 1 day. Mean normalized expression levels (MNE) of Stearoyl-CoA desaturase (*SCD*) (**A**), fatty acid-binding protein 4 (*FABP4*) (**B**), melanocortin receptor 1 (*MC1R*) (**C**) and melanocortin receptor 5 (*MC5R*) (**D**). Stimulation with α-MSH tends to increase gene expression levels of SCD and *MC5R* but not of *MC1R* and *FABP4*. Data are n = 6 (**A**,**B**) n = 3 (**C**,**D**), mean ± SEM.

The OSCs of six different *mice* (3 male/3 female *mice*) were treated with medium containing up to 1000 nM MCR agonist (α-MSH) with/without 1000 nM MCR antagonist (JNJ-10229570) immediately after slicing. After 1 day gene expression levels of two lipogenesis markers (fatty acid binding protein 4, *FABP4* and Stearoyl-CoA desaturase, *SCD*) as well as *MC1R* and *MC5R* were determined (Figure 6). *SCD* plays an important role in the lipid biosynthesis of monounsaturated fatty acids, as it is the rate-limiting enzyme. *SCD* gene expression tended to increase after the addition of 10–1000 nM α-MSH, but not when 1000 nM MCR antagonist (JNJ-10229570) was simultaneously added (Figure 6A). In contrast, *FABP4*, which is known as an intracellular lipid chaperone regulating lipid transport, was not affected (Figure 6B). Although the gene expression of *MC1R* tended to be increased after the addition of 10 nM α-MSH, no trend was apparent (Figure 6C). Gene expression of *MC5R* showed a tendency to increase after the addition of α-MSH. However, this was also the case with the simultaneous addition of 1000 nM MCR antagonist (JNJ-10229570) (Figure 6D).

## 3. Discussion

### 3.1. The Ex Vivo 3D Organotypic Culture of Mouse Meibomian Glands Is Suitable to Study (Patho-) Physiological Properties in Meibomian Glands

Besides the *human*-derived ihMGECs [33], other cell culture systems from rabbits [34] and *mice* [35,36] are available to study MGD. However, in the last decade, these cell culture systems have raised some concerns because they cannot represent the physiologic in vivo situation of a cell dying by holocrine secretion [24]. Moreover, the architecture of MG acini is very complex, making recent attempts to establish a suitable 3D in vitro MG culture model difficult and fraught with limitations [37]. In addition, various animal models [38,39,40,41,42,43,44,45,46,47] and a recently developed *mouse* explant culture [30] are used in MGD. In both cases, the 3Rs concept of directive 2010/63/EU should be considered. Since we cut the OSCs into thin slices of only 150 µm, an average of 30–40 OSCs are produced from one *mouse*, which makes our culture model consistent with the 3R concept. However, the thin slicing of the OSCs has another advantage: In spheroids of epithelial cells, denser cell packing leads to a significant decrease in oxygen supply and thus to necrotic areas within [48]. Poor oxygen supply may therefore be the reason why the cell viability decreases after 72 h in a *mouse* explant model [30] and another in vitro slice culture model of *human* eyelids was only used for 24 h [28]. We, on the other hand, observed no impairment in viability for at least 7 days and were thus the first to culture eyelid tissue over a longer period.

Serum containing media induces lipid accumulation in ihMGECs [33,49,50] and in a *mouse* explant model [30]. In our newly developed OSC of mMGs, the same serum-containing medium results in a steady increase of lipids for up to 21 days and shows that the physiological function of lipid production is preserved. The treatment of ihMGECs with serum leads not only to lipogenesis but also to dramatic changes in morphology by the formation of desmosomes, CK-filaments, and lamellar bodies [49]. The treatment of OSCs with serum led to differentiation of basal meibocytes accompanied by enlargement of the cells, lipogenesis and pyknosis of the cell nucleus. An emergence of new basal meibocytes from the acinar periphery was not observed, thus leading to a kind of stem cell exhaustion. However, we hypothesize that appropriate adaptation of the cultivation medium could preserve basal meibocyte formation, as serum inhibits the proliferation of primary *human* meibomian gland epithelial cells [33].

CK14 has higher expression in *human* basal cells [23,31], but can be stained throughout the glandular acini in *mice* [30]. Accordingly, CK14 antibody reactivity was observed in basal, differentiating, and mature meibocytes in mMG OSCs. E-cad is expressed both in basal and mature meibocytes in *humans* [51]. To date, there is no information about E-cad expression in mMGs. We were able to detect E-cad in basal, differentiating, and mature meibocytes in the OSCs, but with a higher expression in basal cells. The decrease in E-cad immunoreaction after 14 days together with the observation that OSCs occasionally showed detachment may indicate that cell-cell contacts decrease between 7 and 14 days. However, since the decrease in E-cad reactivity is mainly observed in basal meibocytes, it could also be caused by the differentiation of these cells and the simultaneous stem cell exhaustion in the OSCs, as shown previously. Nonetheless, the expression of both biomarkers was maintained for up to 7 days, which, together with the perseverance of viability and physiological function, indicates that the long-term cultivation of mMG OSCs is possible.

### 3.2. Melanocortins and Their Receptors Induce Lipid Production in Meibomian Glands

The melanocortin hormone system regulates a variety of physiological functions including melanogenesis, inflammation, immune modulation, adrenocortical steroidogenesis, hemodynamics, natriuresis, energy homeostasis, sexual function, and exocrine secretion [28,52,53,54,55,56,57,58,59,60]. All five MCRs show different tissue distribution and bind α-MSH, except MC2R, the ACTH receptor [9]. For this reason, MC2R was excluded from this study. Our data reveal that MC1R, MC3R, MC4R and MC5R are expressed in both male and female mMGs, while only MC1R and MC5R are expressed in *human* sebaceous glands [10,11,14]. In sebaceous glands, α-MSH upregulated lipid production as well as MC1R and MC5R expression levels [14,15], while an MCR antagonist, JNJ-10229570, inhibited sebaceous lipid production [16]. Likewise, stimulation with α-MSH induces dose-dependent lipid accumulation in our OSCs, whereas simultaneous treatment with the MCR antagonist inhibits this effect. However, stimulation tends to only induce *MC5R* but not the *MC1R* gene expression in our OSCs. Due to the small size of the OSCs, the fatty nature of the meibomian glands and the interfering agarose, RNA extraction can certainly be improved in our system. In any case, our data provide evidence that melanocortins likely stimulate meibum production and thus prove the physiologic function of our culture system. 

## 4. Conclusions

Our results demonstrate that the viability, physiological function, morphology, and expression of cell markers of mMG were maintained for at least 7 days in our ex vivo OSC culture model. In addition, this study demonstrates for the first time that α-MSH induces lipid accumulation in MGs. Thus, the ex vivo organotypic slice culture model is a promising approach to study (patho-) physiological properties of MG and can be used to accelerate the exploration of new treatments for MDG/DED.

## 5. Materials and Methods

### 5.1. Mice

Male and female C57BL/6J *mice* were obtained from Charles River laboratories (Wilmington, MA, USA) and kept under a 12:12 h light:dark cycle with food and water ad libitum. Tarsal plates with MGs were isolated from the eyelids of 10–16 week old *mice* sacrificed by cervical dislocation. All experiments were conducted in accordance with the ARVO Statement for the use of animals in ophthalmic and vision research and the national ethical committee for animal experimentation (FELASA) and are approved and registered by the Animal Welfare Office of Friedrich-Alexander University (TS-12/14 Anatomie II, 26 August 2020).

### 5.2. Vibratom Slicing and Culture Conditions

The upper eyelids were excised, and the overlying fat and connective tissue were thoroughly removed. The tarsal plates were embedded in 6% (wt/vol) low melting agarose (Bio&Sell, Feucht/Nuremberg, Germany) at 37 °C dissolved in phosphate-buffered saline (PBS, pH 7.3). Immediately after embedding, the agarose was placed on ice to ensure fast curing. The agarose was then cut into a diamond shape for optimal cutting performance and glued onto the specimen holder. The embedded tarsal plates were cut in 150 µm slices at a feed rate of 0.8 mm/s with an amplitude of 1.0 mm using a vibratome (VT1200S Leica, Wetzlar, Germany). After sectioning, the OSCs were immediately transferred to culture medium (Dulbecco’s Modified Eagle’s medium and Ham’s F12 (ratio 1:1; Biochrom, Berlin, Germany), 10% fetal calf serum (Gibco Life Technologies, Karlsruhe, Germany), and 10 ng/mL epidermal growth factor (Thermo Fisher Scientific, Waltman, MA, USA), 1% Penicillin-Streptomycin (Sigma-Aldrich, Taufkirchen, Germany) and cultivated at standard conditions.

For stimulation of MCRs, the OSCs were transferred to a culture medium containing 10–1000 nM α-MSH (Sigma-Aldrich, Taufkirchen, Germany) with/without 1000 nM MCR antagonist JNJ-10229570 (Sigma-Aldrich, Taufkirchen, Germany) and cultivated for up to seven days, and the medium was renewed every two days.

### 5.3. Viability Assay

A viability assay on OSCs was performed according to the manufacturer’s instructions with CellTiter-Glo^®^ 3D Reagent (Promega, Walldorf, Germany) using a CLARIOstar Plus (BMG Labtech, Ortenberg, Germany) plate reader for luminescence measurement. The surface of each OSC was determined using Image J 1.53a (National Institutes of Health, Rockville Pike, MD, USA [61]) to normalize the luminescence signal to the respective size of the OSC.

### 5.4. Live-Dead Assay

The OSCs were incubated for 5 min with 3 µM propodium iodide (Sigma-Aldrich, Taufkirchen, Germany) and 3 µM calcein AM (Sigma-Aldrich, Taufkirchen, Germany) dissolved in PBS. Fluorescence was observed under a fluorescent microscope (Keyence BZ-X800E, Osaka, Japan). The percentage of live and dead cells in MGs relative to the OSC was determined using Image J. Viability was calculated based on the percentage of live cells compared to the total number of cells.

### 5.5. Lipid Quantification

OSCs were incubated with 5 µM Lipi Red Solution (Dojindo Laboratories, Kumamoto, Japan) dissolved in PBS for 30 min at 37 °C according to the manufacturer’s instructions. Fluorescence microscopy was used to analyze the lipid proportion of the MGs. Lipid intensity was measured by Image J. In addition, a bright field image was taken to identify the surface of the MGs.

### 5.6. Histological and Immunohistological Staining

OSCs were fixed with 4% PFA and pre-embedded horizontally in HistoGel™ (Thermo Fisher Scientific, Waltman, MA, USA). The OSCs were then embedded in paraffin with a tissue tamper to ensure horizontal orientation and cut into 5 µm sections. Morphological changes during cultivation were investigated by hematoxylin and eosin (HE)-staining following standard protocols.

For immunohistochemistry, sections were deparaffinized, rehydrated in a descending alcohol series, and treated with 3% H_2_O_2_ (hydrogen peroxide) for 15 min. Antigen retrieval was accomplished by steaming the sections in a 10 mM citrate buffer (pH 6.0) for 35 min. Unspecific binding was blocked using 10% normal goat serum (Agilent, Santa Clara, CA, USA) in TBS-T (Tris-buffered saline with Tween 20). Sections were then treated with an Avidin/Biotin Blocking System (BioLegend, San Diego, CA, USA) according to the manufacturer’s instructions before primary antibodies were applied overnight at 4 °C (Table 1). Afterwards, the sections were incubated with the corresponding biotinylated secondary antibody (Table 1) for one hour, followed by incubation with a VECTASTAIN^®^ Elite ABC-HRP Kit (Vector Laboratories, Burlingame, CA, USA) according to the manufacturer’s instructions. Finally, sections were stained with AEC+ High Sensitivity Substrate (Agilent, Santa Clara, CA, USA) and counterstained with hematoxylin.

### 5.7. Transmission Electron Microscopy

OSCs were fixed with ITO-fixing solution (25% paraformaldehyde, 25% Glutardialdehyde, 0.1 M cacodylatbuffer) and pre-embedded horizontally in HistoGel. The OSCs were then processed for Epon embedding following standard protocols. Ultrathin cross sections were cut with a microtome (Ultracut Leica, Jena, Germany) and analyzed with a JEM-1400Plus (JEOL, Tokyo, Japan). SightX-Viewer software (Jeol, version 1.2.3.537) was used to evaluate the morphological changes.

### 5.8. RNA Extraction, cDNA Synthesis and Reverse-Transcription PCR

Total RNA from *mouse* upper eyelids or OSCs was extracted and transcribed into cDNA as described previously [49]. To ensure cDNA integrity, *mouse* β-actin PCR was carried out for all cDNAs. Each reaction was performed using Taq DNA Polymerase (Thermo Fisher Scientific, Waltman, MA, USA), with 100 mM dNTPs (Thermo Fisher Scientific, Waltman, MA, USA) and 2 × 10 pmol gene-specific primers (Table 2) and run according to manufacturer’s instructions. The PCR products were run alongside a peqGOLD Low Range DNA Ladder (VWR, Darmstadt, Germany) on a 2% agarose gel stained with GelRed Nucleic Acid Gel Stain (Linaris, Dossenheim, Germany).

### 5.9. Quantitative Real-Time PCR (qPCR)

Analysis of gene expression was performed by quantitative real-time PCR using a LightCyclerR 480 (Roche, Basel, Switzerland) in combination with Takyon™ MasterMix (Eurogentec, Seraring, Belgium) according to the manufacturer’s instruction. Amplification was carried out in duplicates of three biological replicates and no-template-sample served as the control. Primer efficiency was calculated for each primer used (Table 2), and data were analyzed according to Pfaffl [62].

### 5.10. Statistics

Data was expressed as mean values with standard deviation. Statistics were performed using GraphPad Prism 9 (version 9.3.0., GraphPad Software Inc., San Diego, CA, USA). Statistical significance was set at a *p* value of ≤ 0.05.

## Figures and Tables

**Table 1:** Antibodies.

Antibody	Conjugation	Solution	Product No.	Manufacturer
Cytokeratin 14	-	1:200	NCL-LL002	Novocastra Laboratories, Newcastle upon Tyne, UK
Goat Anti-*Mouse* IgG	Biotin	1:200	E0433	Agilent Technologies, Santa Clara, CA, USA
E-cadherin (24E10)	-	1:200	3195S	Cell Signaling Technology, Danvers, MA, USA
Goat Anti-Rabbit IgG	Biotin	1:200	E0432	Agilent Technologies, Santa Clara, CA, USA

**Table 2:** Primer sequences.

Primer	Forward	Reverse	Amplicon
*FABP4*	TGA AAT CAC CGC AGA CGA CA	ACA CAT TCC ACC ACC AGC TT	141 bp
*MC1R*	CGC TTC CTA CTT CCT GAC AAG	TCA CAA CCA GCA CAT TCT CC	199 bp
*MC1R*	TGA AGT GAA TCA GAA GCT GGG	AAG GTG AGA GGT GGC ATT G	146 bp
*MC3R*	CCA CAA GAG AAG CAC CTA GAA G	AGC ATC GGA GAA ACA GAA GAC	133 bp
*MC4R*	GAC CCT CTC ATT TAT GCC CTC	AGC TGT TGG GAA GTA CAC TG	191 bp
*MC5R*	AGA TTC AAC TCC CAG AAA CCG	AGA TTC AAT ACA GTC AGG GTG G	149 bp
*RPS6*	CTT TTT CGT GAC GCC TCC CA	GGG AAG GAG ATG TTC AGC TTC A	62 bp
*SCD*	CTG ACC TGA AAG CCG AGA AG	AGA AGG TGC TAA CGA ACA GG	147 bp
*β-actin*	GAT CCT CAC CGA GCG CGG CTA CA	GCG GAT GTC CAC GTC ACA CTT CA	298 bp

*FABP4*: fatty acid binding protein 4; *MC1R*: melanocortin receptor 1; *MC3R*: melanocortin receptor 3; *MC4R*: melanocortin receptor 4; *MC5R*: melanocortin receptor 5; *RPS6*: ribosomal Protein S6; *SCD*: Stearoyl-CoA desaturase.

## Supplementary Materials

The following supporting information can be downloaded at: https://www.mdpi.com/article/10.3390/ijms232314947/s1.
