# Supplementary material for: A New Organotypic 3D Slice Culture of Mouse Meibomian Glands Reveals Impact of Melanocortins
Source: Int J Mol Sci. 2022 Nov 29;23(23):14947. doi: 10.3390/ijms232314947 (PMC9737810; doi:10.3390/ijms232314947)
Supplement: Supplementary file 1 [file ijms-23-14947-s001.zip › Captions Supplemental Figure_Zahn et al..docx]

Figure S1. Viability of organotypic slice cultures (OSC) of *mouse* meibomian glands (mMG) over 21 days treated with 40% DMSO. Viability of mMG OSCs treated with/without 40% DMSO are investigated over 21 days. Treatment of OSCs with 40% DMSO resulted in a considerable decrease in viability on all days (A). Representative pictures of mMG OSCs after live-dead staining illustrates the location and distribution of the live (green) and dead (red) cells. Bright field imaging shows the position of the mMGs within the OSCs. Treatment of OSCs with 40% DMSO resulted in no live cells, but dead cells on all days (B). Data are n = 3, mean ± SEM. Scale bar: 200 µm (B).

Figure S2. Composition of the organotypic slice cultures (OSC) of *mouse* meibomian glands (mMG). Representative bright field image from an OSC on day 0 with cilia, meibomian glands, conjunctiva, tarsus and orbicularis oculi muscle highlighted. Scale bar: 200 µm.

Figure S3. Lipid production of organotypic slice cultures (OSC) of *mouse* meibomian glands (mMG) over 21 days treated with 40% DMSO. Evaluation of fluorescence intensity in the meibomian gland (A), the surrounding tissue (B) and the whole OSC (C) native and treated with 40% DMSO as a negative control. The fluorescence intensity was normalized to day 0. Under DMSO treatment, fluorescence intensity was significant decreased after day 14 and day 21 in the whole OSC and the mMG. Data are n = 4, mean ± SD, * p ≤ 0.05, ** p ≤ 0.01.

Figure S4. Influence of α-MSH and JNJ-10229570 on lipid production organotypic slice cultures (OSC) of *mouse* meibomian glands (mMG) at day 3 and 7 days. No influence on relative lipid production of OSCs treated with medium containing up to 1000 nM α-MSH with/without 1000 nM JNJ-10229570 for 3 days (A) and 7 days (B). Data are n = 4, mean ± SD.
