# Supplementary figures and images for: A New Organotypic 3D Slice Culture of Mouse Meibomian Glands Reveals Impact of Melanocortins
Source: Int J Mol Sci. 2022 Nov 29;23(23):14947. doi: 10.3390/ijms232314947 (PMC9737810; doi:10.3390/ijms232314947)

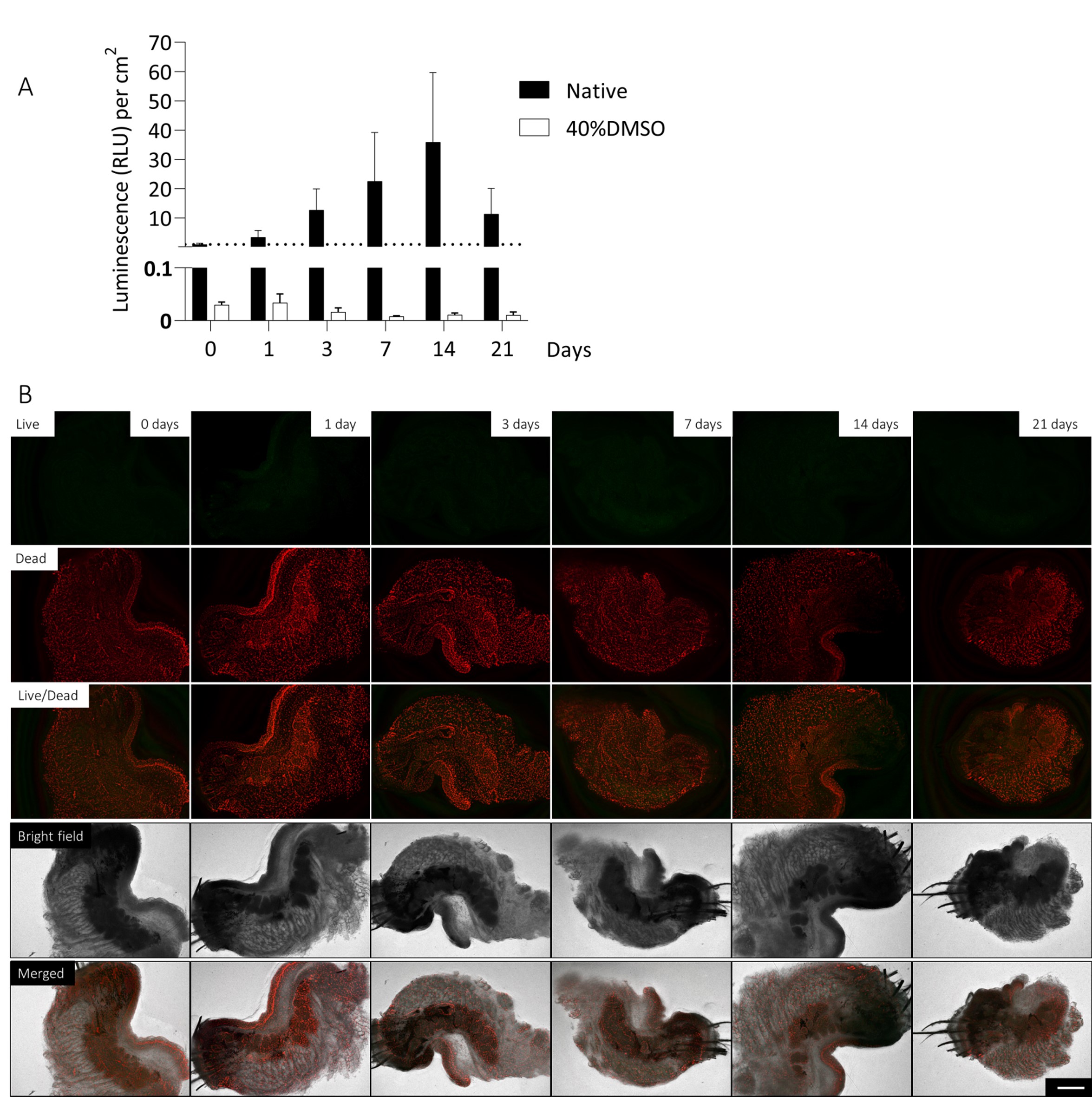

Supplement: Supplementary file 1 [file ijms-23-14947-s001.zip › Supplemental Figure1_Zahn et al..jpg]

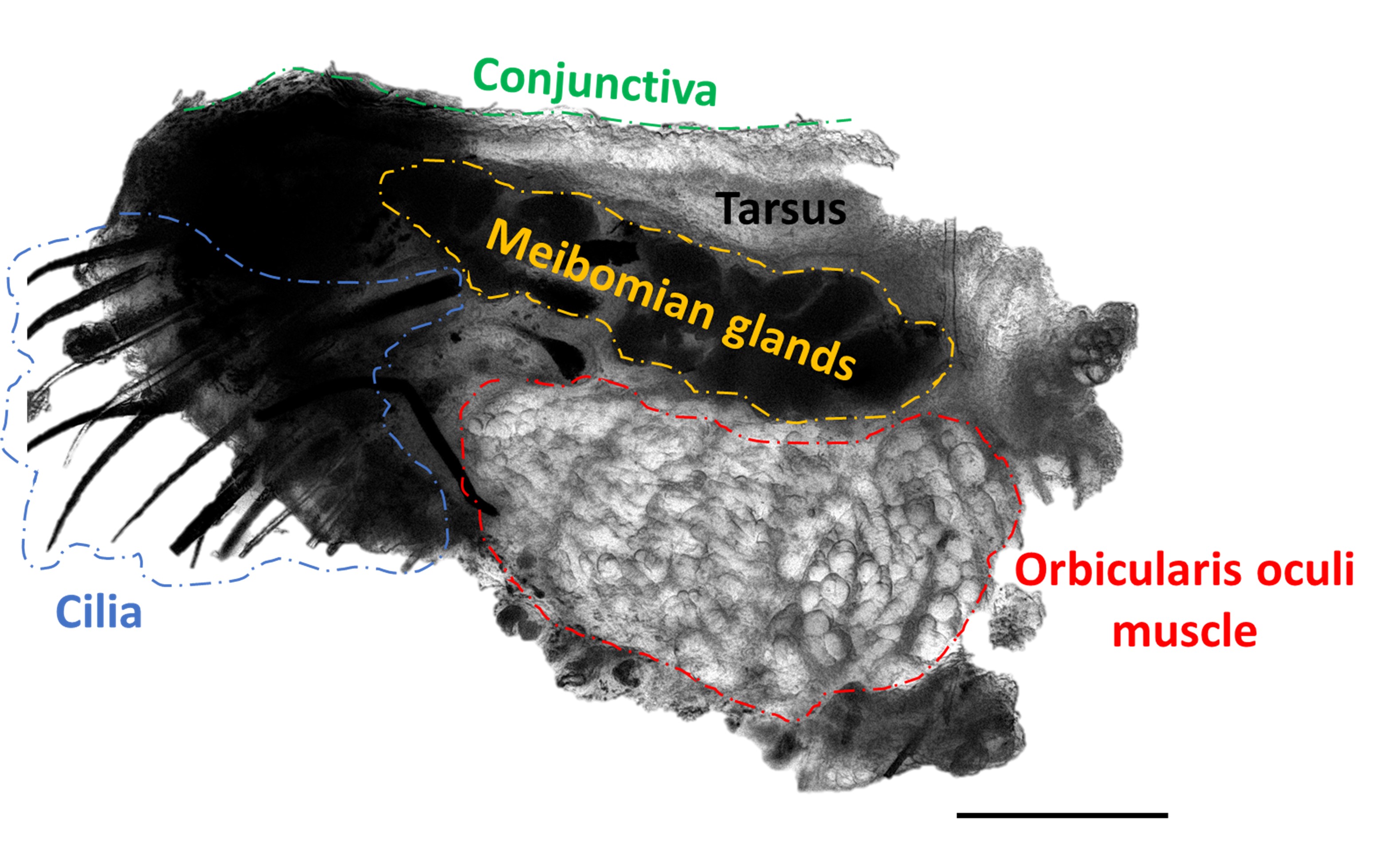

Supplement: Supplementary file 1 [file ijms-23-14947-s001.zip › Supplemental Figure2_Zahn et al..jpg]

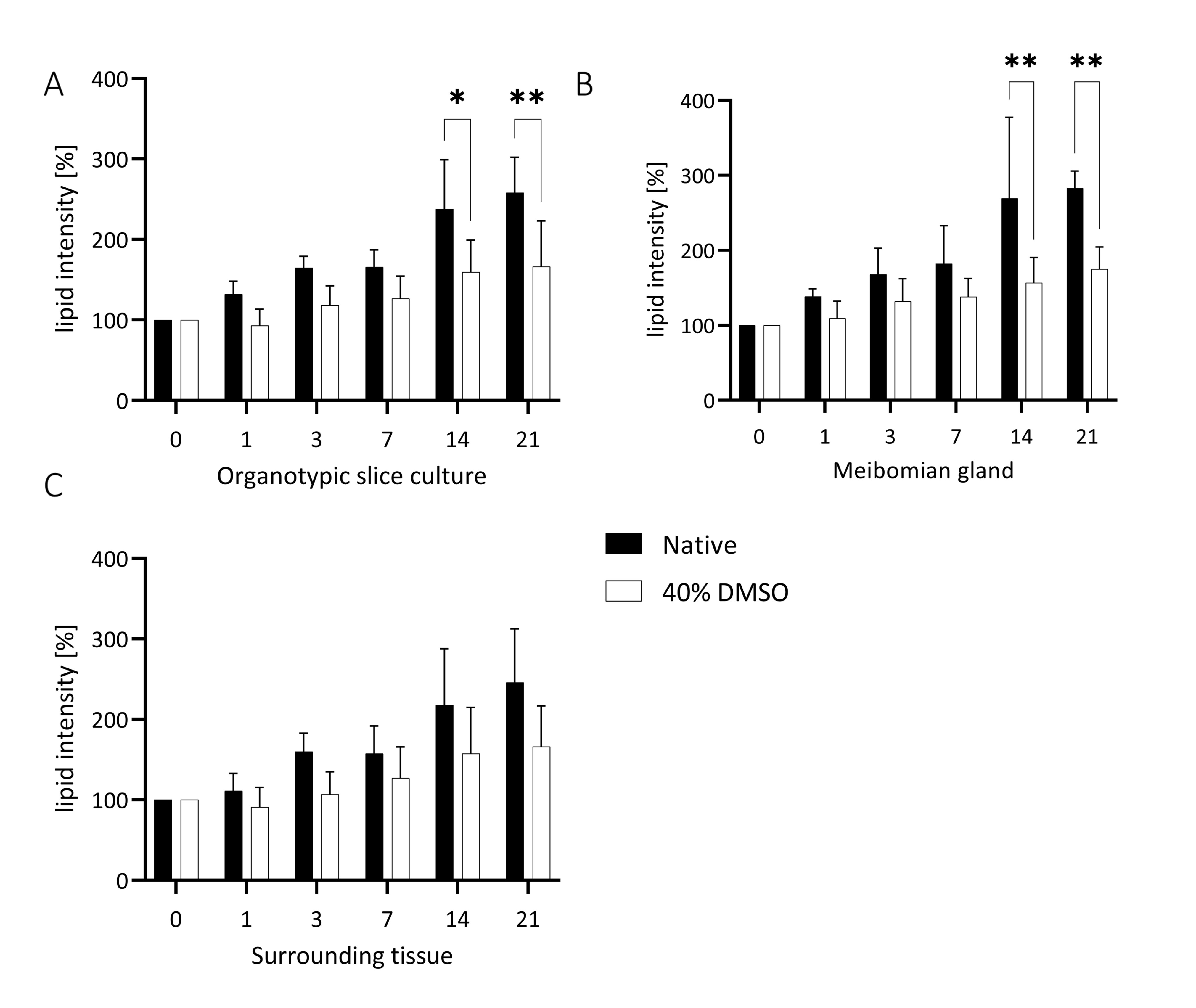

Supplement: Supplementary file 1 [file ijms-23-14947-s001.zip › Supplemental Figure3_Zahn et al..jpg]

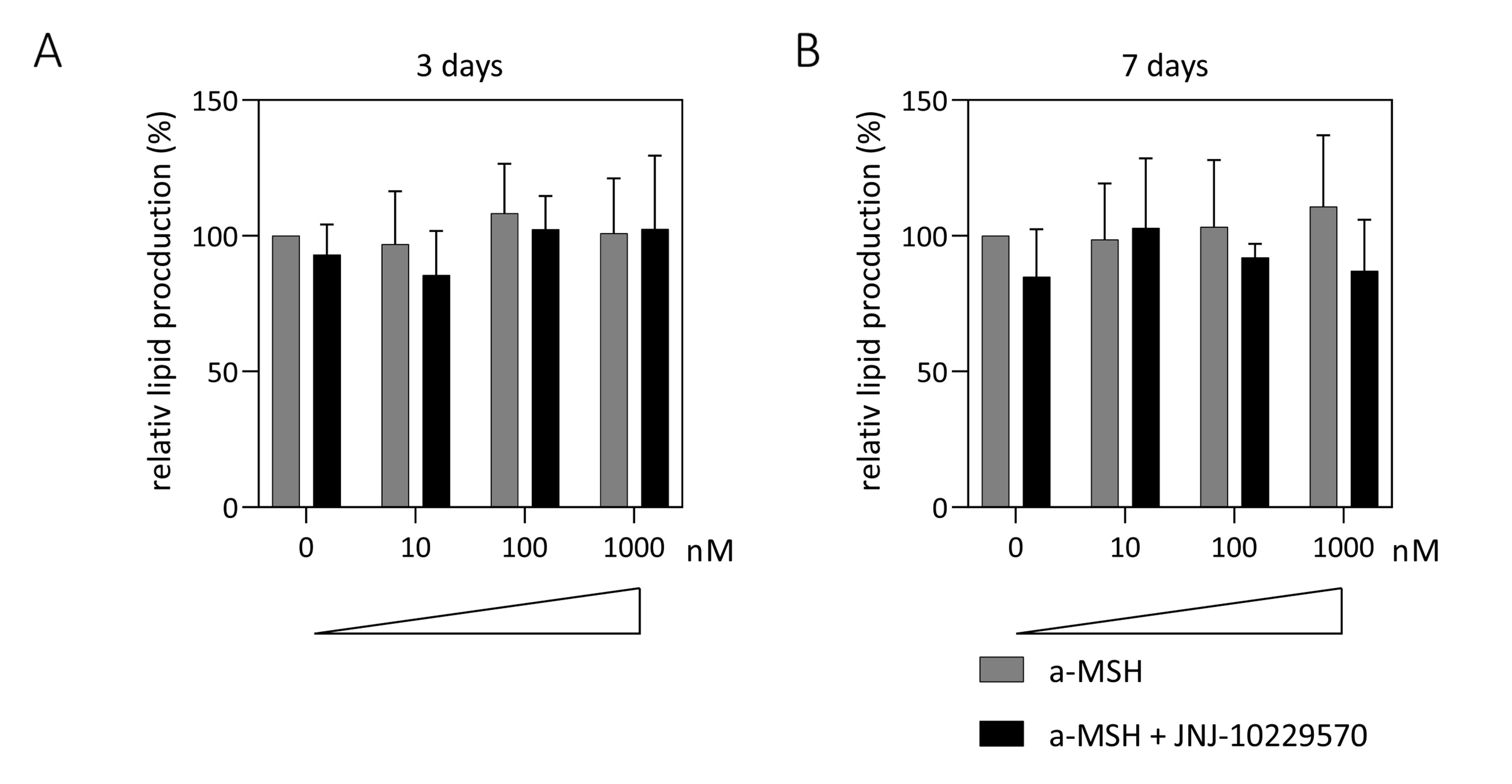

Supplement: Supplementary file 1 [file ijms-23-14947-s001.zip › Supplemental Figure4_Zahn et al..jpg]
